# Murine sterile fecal filtrate is a potent pharmacological agent that exerts age-independent immunomodulatory effects in RAW264.7 macrophages

**DOI:** 10.1186/s12906-023-04193-4

**Published:** 2023-10-13

**Authors:** Bhawna Diwan, Rahul Yadav, Anamika Singh, Dinesh Kumar, Rohit Sharma

**Affiliations:** Faculty of Applied Sciences & Biotechnology, Shoolini University, Solan, 173229 India

**Keywords:** Sterile fecal filtrate, Fecal microbiota transplantation, Macrophages, Inflammation, LPS

## Abstract

**Background:**

Sterile fecal filtrate (SFF) is being considered a safer alternative to fecal microbiota transplantation (FMT) therapy; however, its bioactive potency is very little understood. The present study thus assessed the age-dependent immunostimulatory and immunomodulatory attributes of murine SFF in vitro.

**Methods:**

SFF from young (Y-SFF) and old (O-SFF) Swiss albino mice were prepared. Immunostimulatory and immunomodulatory effects of SFF were evaluated in resting and lipopolysaccharide (LPS) stimulated macrophage cells by measuring intracellular reactive oxygen species (ROS), nitric oxide (NO) production, inflammatory cytokines profile, as well as gene expression of oxidative and inflammatory transcription factors. SFF were also evaluated for native antioxidant capacity by measuring DPPH and ABTS free radical scavenging activity. Bioactive components present in SFF were also determined by GC/MS analysis.

**Results:**

Both Y-SFF and O-SFF induced potent immunostimulatory effects characterized by changes in cell morphology, a significant increase in NO production, ROS levels, and an increased ratio of pro-inflammatory (IL-6, TNF-α, IL-1β) to anti-inflammatory (IL-10) secretory proteins although no significant aggravation in the transcription of *NF-κB* and *Nrf-2* could be observed. Application of LPS to cells significantly augmented a pro-oxidative and pro-inflammatory response which was much higher in comparison to Y-SFF or O-SFF application alone and mediated by strong suppression of *Nrf-2* gene expression. Pre-treatment of macrophages with both Y-SFF and O-SFF robustly attenuated cellular hyperresponsiveness to LPS characterized by significantly decreased levels of NO, ROS, and inflammatory cytokines while a concomitant increase in anti-inflammatory protein (IL-10) was observed. Further, both Y-SFF and O-SFF strongly resisted LPS-induced downregulation of *Nrf-2* expression although O-SFF appeared to protect cells slightly better from the overall LPS threat. Neat SFF samples exhibited moderate antioxidant capacity and GC/MS analysis of SFF revealed diverse volatile organic compounds characterized by alkanes, organosulphur compounds, furans, amides, amino acids, and antimicrobial elements.

**Conclusion:**

Our results indicate that SFF is a potent stimulant of macrophages and confers strong anti-inflammatory effects regardless of donor age thereby suggesting its therapeutic efficacy in lieu of FMT therapy.

## Background

The role and relevance of the gut microbiota in shaping the various facets of human health including immunological maturation, inflammatory response, as well as metabolic health are well recognized [1, 2]. Accumulating evidence suggests that gut microbiota dysbiosis is a key event underlying several metabolic and chronic inflammatory disorders [3–5]. Further, strategies aimed at the restoration of the eubiotic gut have been shown to be effective in alleviating the aggression and severity of inflammation and injury [6, 7]. Supported by these observations, fecal microbiota transplantation (FMT) therapy garnered significant interest in managing gastrointestinal infections [8] as well as chronic inflammatory disorders [9, 10]. FMT involves the transfer of the gut microbiota of a pre-screened healthy donor to a patient with a documented imbalance in gut microbial profile in an attempt to change the recipient’s gut microbiota and ultimately confer health-beneficial effects. Although FMT is generally considered safe and effective; yet, concerns related to its applicability in specific conditions such as in immunocompromised adults and infants, its long-term efficacy, as well as its tendency to induce latent or novel infections, are yet to be completely understood and resolved [9, 11].

FMT does not simply involve the transfer of live microbes, but a considerable number of dead bacteria and their metabolites are also transferred in the process which are likely to contribute to the benefits associated with FMT [12]. Considering this as well as the safety limitations of FMT, it was demonstrated that the transfer of sterile fecal filtrate (SFF) from healthy donors alone was sufficient to relieve the symptoms of *Clostridium difficile* infection [13]. Similarly, a recent study showed that SFF treatment could be effective in preventing infection in cesarean-delivered piglets as models for preterm infants [14]. These studies thus indicate that if the efficacy of SFF is anywhere comparable to FMT therapy, it may present a safer and more amenable therapeutic approach than FMT for managing gut inflammatory disorders and maintaining homeostasis of the gut microenvironment. However, the extent and depth of the therapeutic potency of SFF are least explored and are only beginning to be understood. Given that FMT therapy has already demonstrated anti-inflammatory effects, it would be interesting to assess whether SFF can also confer such cellular and immune regulatory attributes. Therefore, in the present work, we sought to assess the immunoregulatory and pharmacological attributes of murine derived SFF in vitro in an attempt to ascertain its therapeutic efficacy. In addition, we also tested the effects of donor mice age on the potency of SFF to establish any age-dependent correlation.

## Methods

### Animal husbandry

Young (4 months) and old (20 months) male Swiss albino mice were procured from the animal house facility of CSIR-IHBT, Palampur. Animals were divided into two groups (Young and Old) of six mice each and were maintained in the animal experiment facility at Shoolini University, Solan. All animals were kept under standard experimental conditions (12:12 h reversed light/dark cycle; relative humidity at 50–60%, temperature of 22 ± 2 °C, and adequate ventilation) and were fed on a commercial animal diet. This particular species, age, and sex of animals were chosen based on our previous experience wherein these animals display characteristic markers of immunosenescence beginning at the age of 16 months [15]. All animal experiments were conducted as per guidelines and approval of the institutional animal ethics committee of Shoolini University, Solan (Approval no. IAEC/SU/21/10).

### SFF preparation

Fecal matter of young and old animal groups was separately collected and weighed in the morning hours (~ 9.00 am IST) for seven consecutive days. Subsequently, it was transferred to phosphate-buffered saline (PBS; pH 7.4) (at 50 mg feces per mL) and homogenized on ice to make a thin slurry [16]. The obtained fecal slurry was centrifuged at 1,000 × *g* for 15 min at 4 °C following which the supernatant was carefully collected. The fecal supernatant was first filtered through a disposable filter paper (Whatman, pore size 4–12 μm) and then through a 0.22 μm filter to finally obtain the sterile fecal filtrate (SFF) for young (Y-SFF) and old (O-SFF) animal groups. The SFF was aliquoted and immediately stored at -80 °C till further analyses. No animal sacrifice was necessary for this work.

### Cell lines and treatment protocol

RAW264.7 murine macrophage cells were obtained from the National Centre For Cell Science (NCCS), Pune, India. The cell lines were cultivated and maintained in Dulbecco’s Modified Eagle’s Medium (DMEM, AT151; HiMedia, India) supplemented with 10% fetal bovine serum and 100 μg/mL of penicillin–streptomycin (15,140,122; Invitrogen, USA) at 37 ºC in a 5% CO_2_ incubator. For immunostimulation studies, RAW264.7 cells were exposed to different concentrations of Y-SFF and O-SFF for 48 h followed by an assessment of various cellular and biochemical markers. This relatively longer duration of stimulation was chosen to understand the chronic effects of SFF exposure that could also be implicated in its overall safety. For immunomodulatory studies, RAW264.7 cells were first exposed to Y-SFF and O-SFF at respective concentrations for 48 h followed by stimulation with 1 μg/mL of lipopolysaccharide (LPS) (Merck; L2630) for 24 h, and subsequent assessment of various cellular and biochemical parameters. Control cells (C) and LPS-only treated cells were also maintained in parallel.

### Cell viability assay

3-[4,5-dimethylthiazole-2-yl]-2,5-diphenyltetrazolium bromide (MTT) assay was utilized for measuring cell viability in response to SFF treatments. Briefly, cells were seeded in 96-well plates at standardized seeding densities, i.e., at 10,000 cells/well for RAW 264.7 cells; 6,000 cells/well for A549 cells; and 8,000 cells/well for MDA-MB-231 and HepG2 cells and were incubated for 24 h in a humidified CO_2_ incubator at 37 ºC. Cells were then treated with different concentrations of Y-SFF and O-SFF [0.1, 0.20, 0.50, 1, 5, 10, 25, and 50% (v/v)] along with the control. After 48 h of treatment, 10 μL of MTT solution (5 mg/mL) was added to each well, and cells were further exposed for 4 h at 37 °C. Subsequently, the developed formazan crystals were dissolved in 100 μL of DMSO, and absorbance at 570 nm was measured using a Varioskan Lux Microplate Reader (Cat# VL0L00D0; Thermo Fisher Scientific) as per the manufacturer’s instructions. Percent cell viability relative to control was calculated as described previously [17].

### DCFH-DA assay for detection of intracellular reactive oxygen species

Levels of reactive oxygen species (ROS) in RAW 264.7 cells were detected using 2′,7′-dichlorofluorescin diacetate (DCFH-DA) (Merck, D6883) redox probe as previously described [18]. Briefly, cells were seeded in 6-well plates at 20,000 cells/cm^2^ in DMEM for 24 h followed by sample treatment at respective concentrations for 48 h. After incubation, cells were washed with PBS and incubated with DCFH-DA solution (10 μM) for 30 min at 37 °C in a CO_2_ incubator. Cells were again washed twice with PBS and subject to cell lysis using RIPA buffer. The cell lysate was then centrifuged at 16,000 × *g* for 15 min at 4 °C and the supernatant was collected. The supernatant (100 μL) was then transferred to a black 96-well plate and the fluorescence intensity was immediately measured using Varioskan Lux Microplate Reader at an excitation wavelength of 485 nm and an emission wavelength of 530 nm. ROS levels in each sample are expressed relative to fluorescence intensities.

### Nitric oxide production assay

Nitric oxide (NO) levels were quantified in the culture supernatants using the Griess reagent assay kit (Cat.#G2930) (Promega, Madison, WI, U.S.A) according to the manufacturer’s protocol. Briefly, 50 µL of the sample was added to a 96-well plate, followed by the addition of 50 μL of sulfanilamide solution (1% sulfanilamide in 5% phosphoric acid) and subsequent incubation for 10 min at RT, protected from light. Afterward, 50 μL of NED solution (0.1% N-1-napthylethylenediamine dihydrochloride in water) was added to each well, and the mixture was incubated for another 10 min at RT, protected from light. Finally, the absorbance was measured at 540 nm using the Varioskan Lux Microplate Reader. The nitrite concentration in the samples was quantified relative to the standard sodium nitrite.

### Measurement of cytokines by ELISA

RAW 264.7 cells were seeded in a 6-well plate at 20,000 cells/cm^2^ seeding density for 24 h. Cells were then treated with different concentrations of Y-SFF, O-SFF, and/or LPS following which the culture supernatants were collected and stored at -80 ºC until further assessment. IL-6, TNF-α, IL-1-β, and IL-10 cytokines were estimated in culture supernatants using commercially available sandwich ELISA kits (ELISA MAX™ Deluxe set, BioLegend, San Diego, U.S.A) according to the manufacturer’s protocol. Results are expressed as picograms/mL.

### RNA extraction and qRT-PCR

Total cellular RNA was isolated using the TRI-reagent (Sigma-Aldrich, USA, Cat. #T9424). Briefly, after respective treatments, cells were homogenized in TRI-reagent and total RNA was isolated as per the manufacturer’s protocol [19]. The quality and quantity of the isolated RNA were determined, and the RNA was aliquoted and stored at -80 °C until further analysis. qRT-PCR was performed using CFX96 Touch Real-Time PCR Detection System (BioRad Inc.). In brief, 50 ng of RNA template was used per reaction using the Thermo Scientific Verso SYBR Green 1-Step qRT-PCR Low ROX Kit (Cat. #AB-4106/C) as per the manufacturer’s protocol. GAPDH (glyceraldehyde-3-phosphate dehydrogenase) expression was utilized as a housekeeping control to quantify relative mRNA expression using the ΔΔCt method as previously described [19]. Primers used for *Nrf-2* expression: Forward 5-CTGAACTCCTGGACGGGACTA-3’; Reverse 5’-CGGTGGGTCTCCGTAAATGG-3’ and for *NF-κB* expression: Forward 5’-AGCTGATGTGCATCGGCAAGTG-3’; Reverse 5’- GTAGCTGCATGGAGACTCGAACAG-3’.

### DPPH (1, 1-Diphenyl-2-Picrylhydrazyl) free radical scavenging activity assay

The DPPH free radical assay is based on a single electron transfer that produces a violet-colored solution in methanol. This free radical gets reduced in the presence of an antioxidant molecule and thus giving rise to a colorless solution. To determine the DPPH radical scavenging activity of SFF samples, 180 μL of 0.1 mM DPPH (SRL Chemicals, 29,128) solution in methanol was added to 20 μL of the sample in a 96-well plate and incubated in the dark for 30 min. Subsequently, the decolorization of DPPH solution was measured at 517 nm using the Varioskan Lux Microplate Reader. The percentage scavenging activity of the sample was calculated and reported using the formula [20]:

$$\mathrm{Inhibition }(\mathrm{\%}) = [({\mathrm{A}}_{\mathrm{Control}}-{\mathrm{A}}_{\mathrm{Sample}})/ {\mathrm{A}}_{\mathrm{Control}}] \times 100$$

### ABTS [2,2’-Azinobis(3-Ethylbenzothiazoline-6-Sulphonic Acid)] free radical scavenging activity assay

ABTS free radical activity scavenging method is based on the decolorization of ABTS radical cations in the presence of antioxidants in the test sample. ABTS free radical scavenging potential in SFF was determined as per a previous method with slight modifications [21]. A 7 mM solution of ABTS (SRL Chemicals, India; 194,430), was prepared in distilled water to which 2.45 mM potassium persulfate was added. The solution was stored in the dark for 12–18 h for the generation of ABTS radical cation. The ABTS radical cation solution was then diluted with PBS such that it showed an absorbance equivalent to 0.8 ± 0.05 at 734 nm. For performing the assay, 20 μL of the SFF sample and 180 μL of prepared ABTS radical cation solution were added to a 96-well plate. The mixture was then incubated for 10 min at RT, protected from light, and absorbance was measured at 734 nm using the Varioskan Lux Microplate Reader. The percentage scavenging activity of the sample was calculated and reported as per the formula:

$$\mathrm{Inhibition }(\mathrm{\%}) = [({\mathrm{A}}_{\mathrm{Control}}-{\mathrm{A}}_{\mathrm{Sample}})/ {\mathrm{A}}_{\mathrm{Control}}] \times 100$$

### Gas chromatography-mass spectrometry (GC–MS) analyses

GC–MS was performed using gas chromatography (Thermo Fisher Scientific™ TRACE™ 1300 GC)—mass spectrometer (Thermo Fisher Scientific™ TSQ™ Duo triple quadrupole GC–MS/MS) equipped with Triplus RSH-Autosampler. Trace TG-5MS column (40 m length × 0.15 mm inner diameter × 0.15 µm film thickness) was employed to separate out various components. Samples were chemically derivatized with BSTFA (N,O-bis(trimethylsilyl)trifluoroacetamide) and TMCS (Trimethylchlorosilane) in the presence of pyridine for optimum GC–MS performance. Helium was used as a carrier gas with a flow rate of 0.7 ml per min. One microliter sample was injected into the inlet chamber of the GC × GC–MS system in a split-less mode. The column temperature was kept at 60 ℃ for 5 min at the beginning and further allowed to reach 250 ℃ with a ramp rate of 10 ℃ per minute. MS conditions included a transfer line temperature of 250 ℃; ion source temperature at 230 ℃; ionization mode was EI (electron impact ionization); scanning of masses was done from 40–600 units with an elapsed time of 4 min. Identification of peaks in the chromatogram was achieved based on their retention indices and interpretation of mass spectrum by comparing with NIST/EPA/NIH mass spectral library, version 2.2, 2014.

### Statistical analyses

Data are expressed as mean ± S.D (*n* = 3). Significant differences among the groups were determined using one-way ANOVA followed by Tukey’s post-hoc test for multiple comparison corrections. Differences between means were considered statistically significant at *p* ≤ 0.05.

## Results

### Effect of SFF on cell viability

**Fig. 1:**
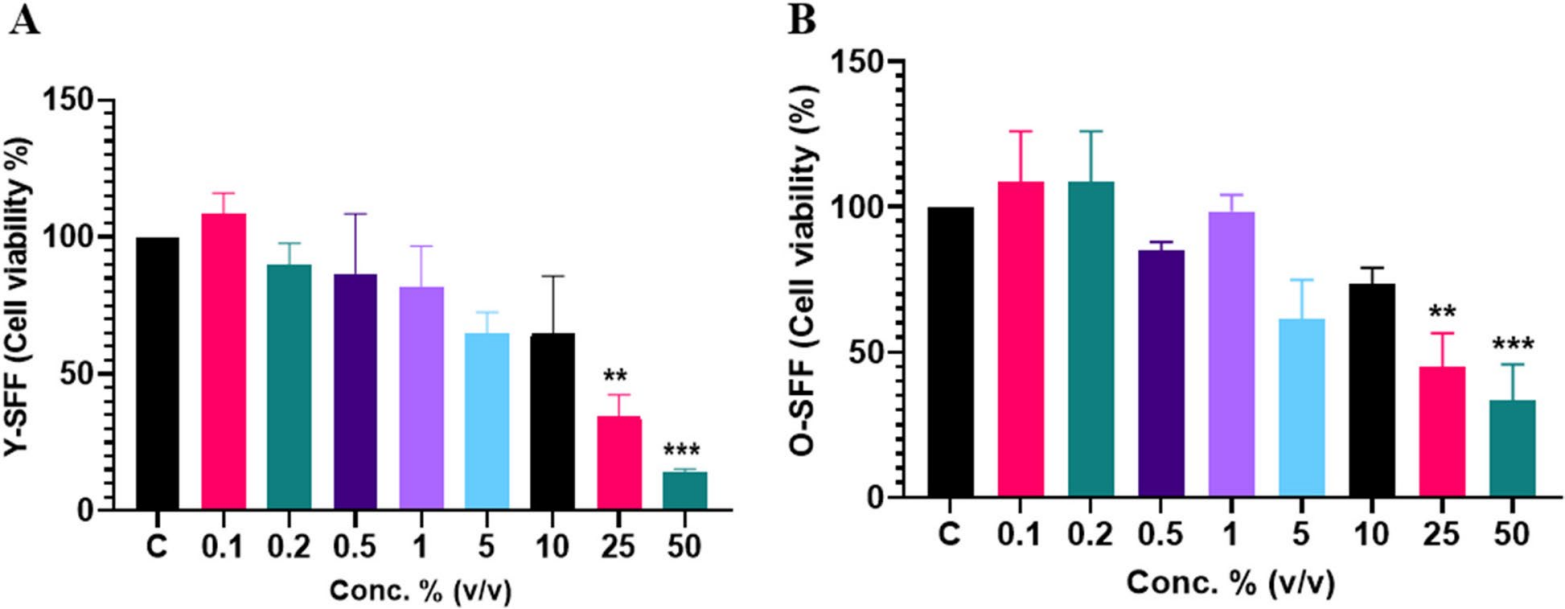
Effect of (**A**) Y-SFF and (**B**) O-SFF on macrophage cell viability at different concentrations. Values are mean ± S.D (*n* = 3). *Represents significant difference as compared to the control group; **p ≤ 0.01, ***p ≤ 0.001

SFF derived from young (Y-SFF) and old (O-SFF) animals were exposed to murine RAW264.7 cells at increasing concentrations. It was observed that low doses of either Y-SFF or O-SFF did not significantly influence cell viability (Fig. 1). However, at higher doses of SFF, i.e., at 25% and 50%, strong evidence of cell death was apparent which reached a maximum of 85.9% in Y-SFF and 66.51% in O-SFF (Fig. 1). Based on these results, the doses of 0.1%, 0.2%, 0.5%, and 1% (v/v) were chosen for both Y-SFF and O-SFF in the subsequent experiments.

### Immunostimulatory effects of SFF

#### Cell morphology

**Fig. 2:**
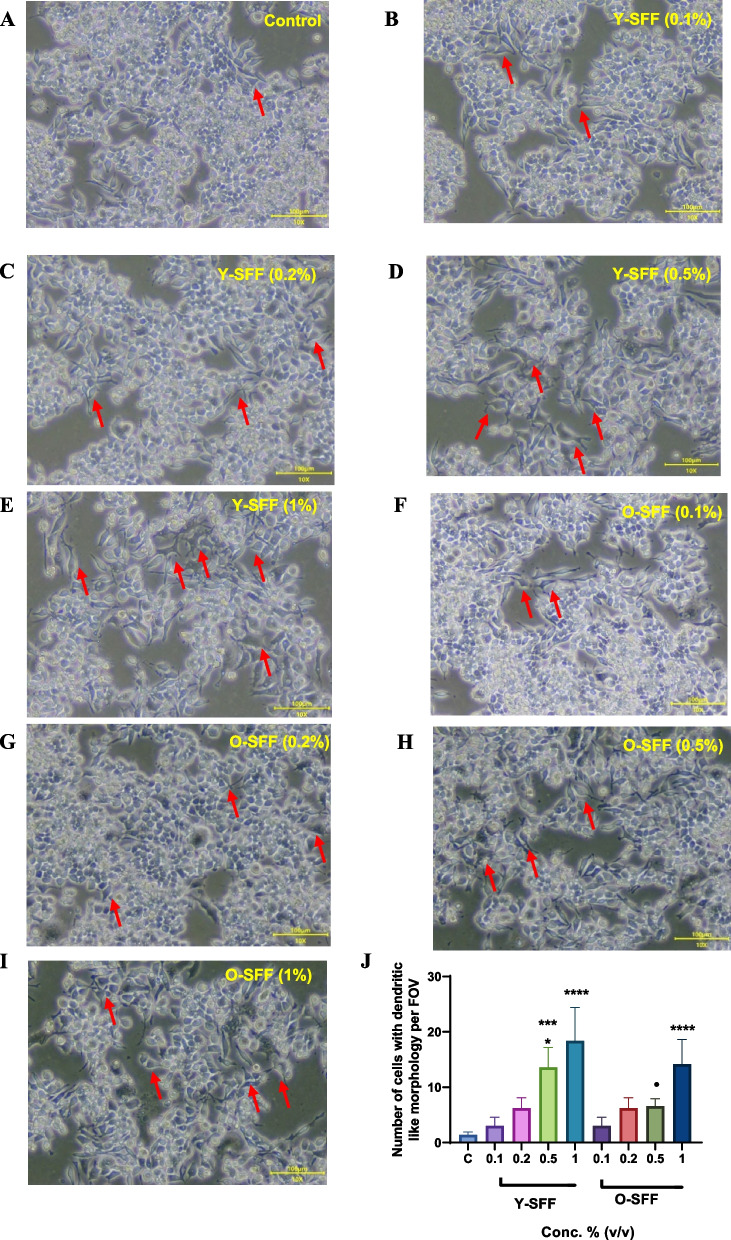
Microscopic images of macrophages showing the effect of SFF (**A**) Control (**B**-**E**) Y-SFF and (**F**-**I**) O-SFF on cell morphology at 10X magnification. **J** Number of cells with dendritic-like morphology per FOV for five regions. Values are mean ± S.D (*n* = 3). *Represents significant difference as compared to the control group; ***p ≤ 0.001, ****p ≤ 0.0001. •Represents significant difference between Y-SFF and O-SFF at the selected mean; •*p* ≤ 0.05

To establish their immune stimulatory attributes, Y-SFF and O-SFF were exposed to RAW264.7 macrophage cells for 48 h followed by a series of cellular and biochemical analyses. The microscopic evaluation indicated dose-dependent stimulatory effects of SFF on macrophages as evidenced by an increase in relative cell size and the appearance of polygonal cells with dendritic-like morphology in comparison to relatively round-shaped control cells as also noted previously [22] (Fig. 2A-I). Further, these effects appeared to be relatively more pronounced in Y-SFF treated cells as compared to O-SFF treated cells (Fig. 2J).

#### Intracellular ROS production

**Fig. 3:**
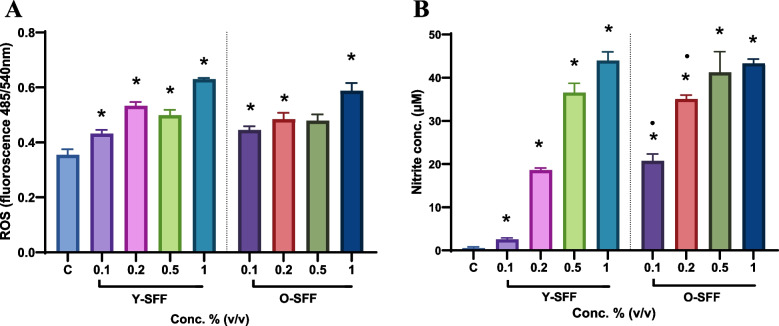
Influence of Y-SFF and O-SFF exposure on (**A**) Intracellular levels of ROS and (**B**) NO production in macrophages at different concentrations. Values are mean ± S.D (*n* = 3). *Represents significant difference as compared to the control group; *p ≤ 0.05, •Represents significant difference between Y-SFF and O-SFF at the selected mean

To establish immunostimulation, cells were analyzed for intracellular ROS production indicative of oxidative burst in macrophages. SFF treated cells at all concentrations exhibited a statistically significant increase in ROS production as compared to the control (Fig. 3A). This effect was significantly more noticeable at the highest tested concentration (i.e., 1% v/v) in both Y-SFF and O-SFF treated cells (Fig. 3A). However, no age-dependent statistically significant effect on ROS levels could be observed although a mildly improved ROS production in Y-SFF treated cells was apparent (Fig. 3A).

#### NO production

NO production by macrophages in response to SFF was evaluated. A robust and significant dose-dependent increase in NO production was observed in all SFF treated cells indicating activation of macrophages (Fig. 3B). Cells treated with low doses of O-SFF induced a statistically significant increase in the production of NO than Y-SFF at similar concentrations; however, no such difference was noticeable at higher concentrations (Fig. 3B).

#### Interleukins production

**Fig. 4:**
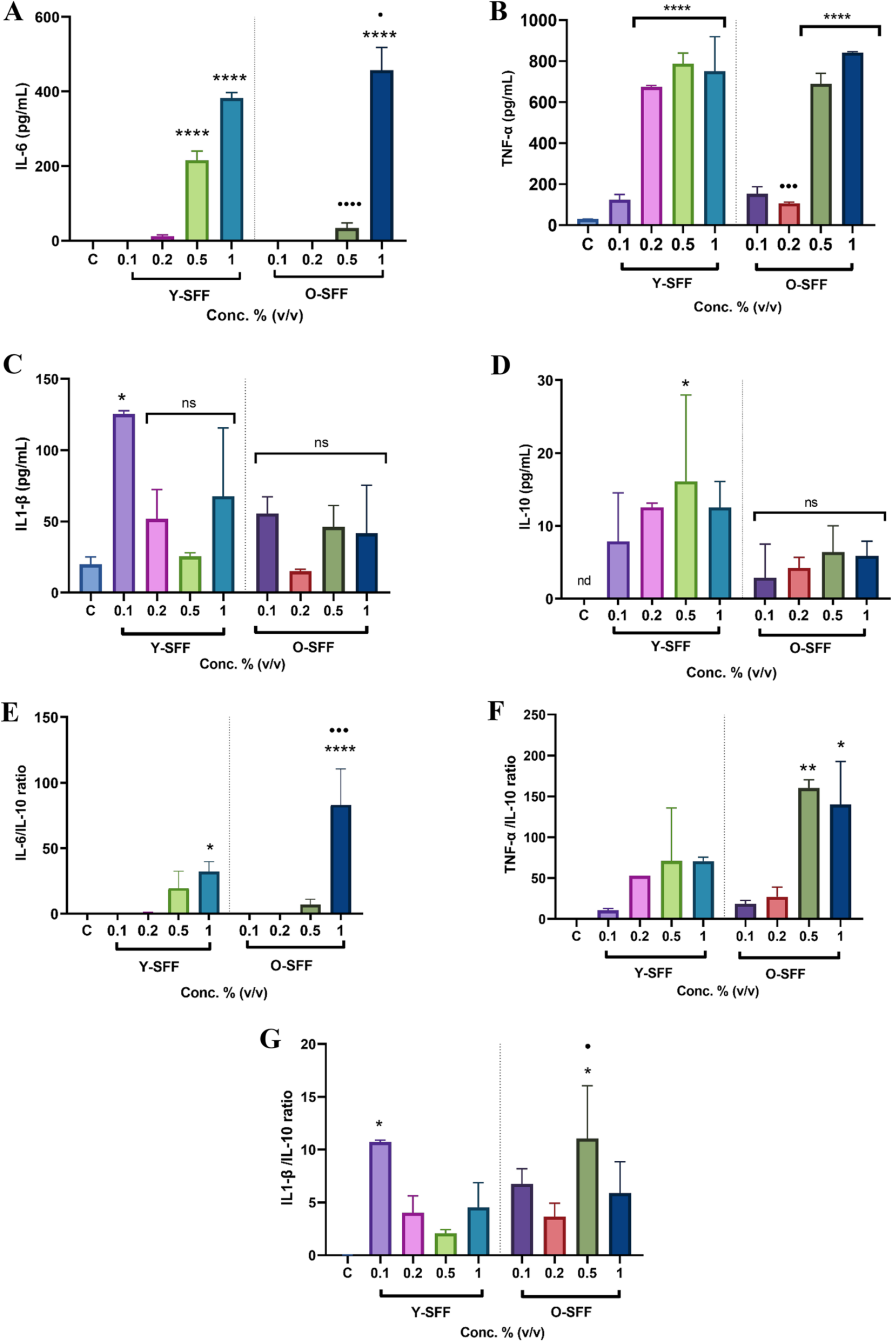
Effect of Y-SFF and O-SFF exposure on interleukins production (**A**) IL-6 (**B**) TNF-α (**C**) IL-1β (**D**) IL-10 (**E**) IL-6/IL-10 ratio (**F**) TNF-α /IL-10 ratio (**G**) IL-1β/IL-10 ratio production in macrophages at different concentrations. Values are mean ± S.D (*n* = 3). *Represents significant difference as compared to the control group; *p ≤ 0.05, **p ≤ 0.01, ***p ≤ 0.001, ****p ≤ 0.0001 •Represents significant difference between Y-SFF and O-SFF at the selected mean; •*p* ≤ 0.05, ••*p* ≤ 0.01, •••*p* ≤ 0.001, ••••*p* ≤ 0.0001

Y-SFF treatment at higher concentrations (0.5 and 1% v/v) robustly and significantly enhanced the production of IL-6 levels in macrophages thereby indicating strong stimulation (Fig. 4A). The effect appeared to be more dose-dependent in Y-SFF treated cells while O-SFF treated cells appeared significantly more responsive to the highest tested concentration (1% v/v) (Fig. 4A). Similarly, TNF-α estimation revealed a significant and relatively stronger response in Y-SFF treated macrophages even at lower concentrations while cells exposed only to the higher concentrations of O-SFF recorded an increase in TNF-α production (Fig. 4B). On the other hand, IL-1β levels in cells were more induced only when exposed to 0.1% concentration (v/v) of both Y-SFF and O-SFF while no dose-dependent increase was observed (Fig. 4C). Although statistically non-significant, levels of anti-inflammatory cytokine IL-10 appeared to be more pronounced in cells treated with Y-SFF as compared to O-SFF treated cells (Fig. 4D). Analysis of the ratio of measured pro-inflammatory cytokines with anti-inflammatory IL-10 clearly indicated that inflammatory homeostasis could be more robustly achieved in Y-SFF treated cells while O-SFF treatment conferred pro-inflammatory effects, especially at higher concentrations (Fig. 4E-G).

### Immunomodulatory effects of SFF

#### Cell morphology

**Fig. 5:**
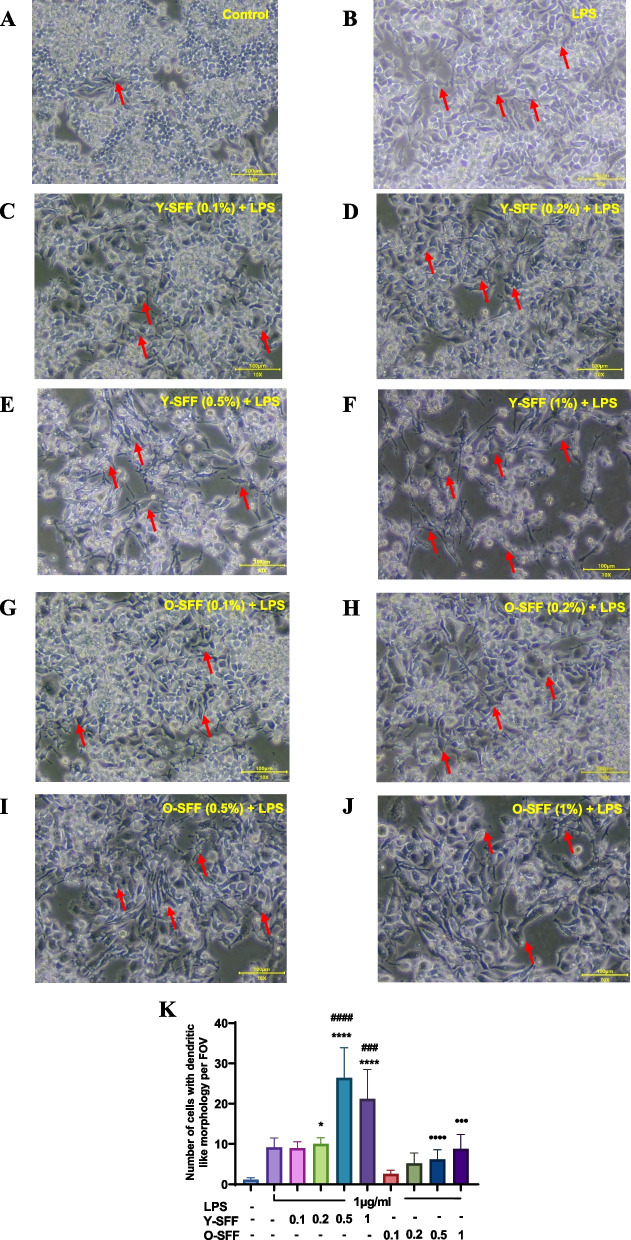
Microscopic images of macrophages showing the morphological effects of LPS and SFF. **A** Control **B** LPS **C**-**F** LPS and Y-SFF at different concentrations **G**-**J** LPS and O-SFF at different concentrations at 10X magnification. **K** Number of cells with dendritic-like morphology per FOV for five regions. Values are mean ± S.D (*n* = 3). *Represents significant difference as compared to the control group; *p ≤ 0.05, •Represents significant difference between Y-SFF and O-SFF at the selected mean; •••*p* ≤ 0.001, ••••*p* ≤ 0.0001. #Represents significant difference as compared to the LPS group at ###*p* ≤ 0.001, ####*p* ≤ 0.0001

To assess whether SFF pre-treatment could modulate the immune response in the wake of an inflammatory threat; an LPS-based cellular model of inflammation was established in RAW264.7 macrophages. Microscopic examination showed clear signs of stimulation in LPS-treated macrophages as evidenced by the appearance of polygonal and dendritic-like morphology and an increase in relative cell size (Fig. 5A-J). Cells pre-treated with SFF showed stronger changes in cell morphology as compared to LPS alone treated cells, and apparent differences in cell morphology on account of Y-SFF and O-SFF application were also noticeable (Fig. 5K).

#### Respiratory burst analyses

**Fig. 6:**
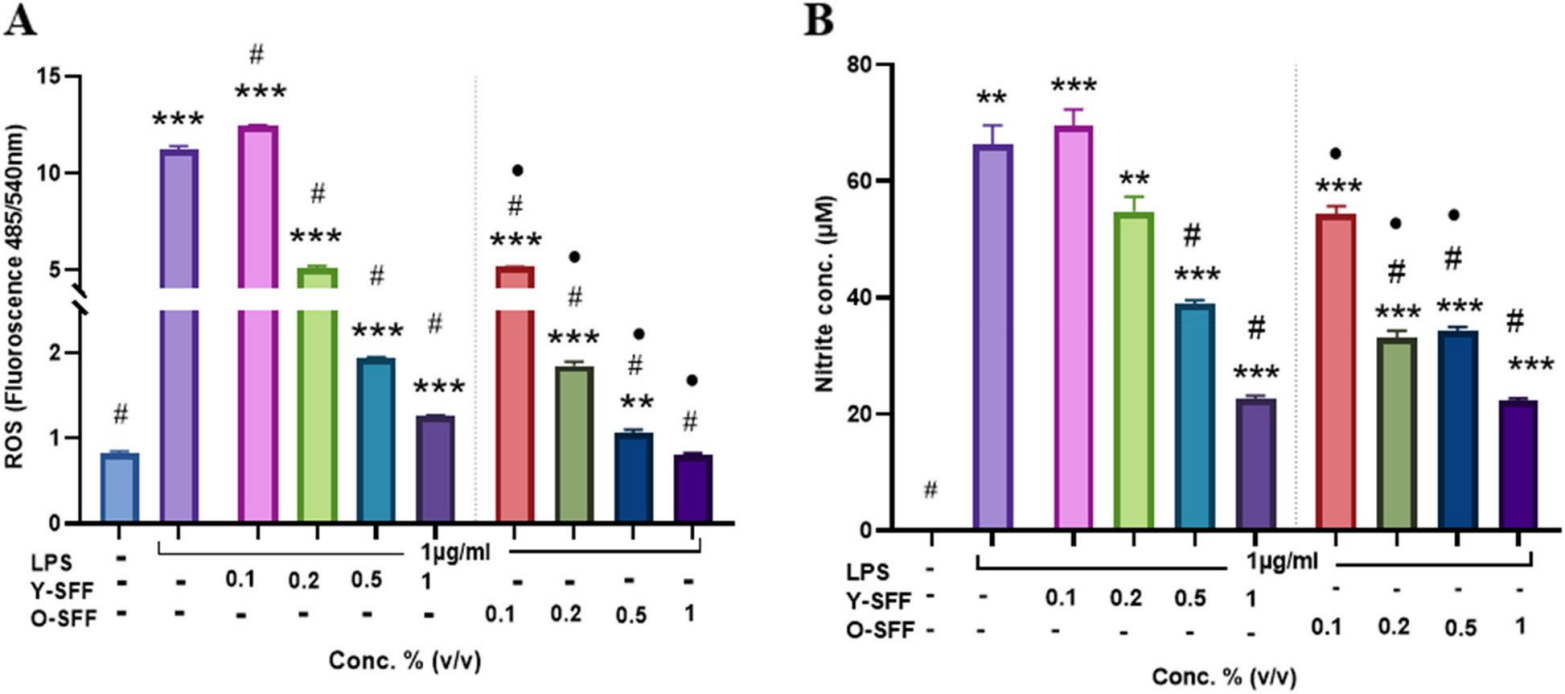
Influence of Y-SFF and O-SFF exposure in attenuating LPS-induced oxidative stress in macrophages (**A**) Intracellular levels of ROS and (**B**) NO production at different concentrations. Values are mean ± S.D (*n* = 3). *Represents significant difference as compared to the control group; *p ≤ 0.05, **p ≤ 0.01, ***p ≤ 0.001, ****p ≤ 0.0001 •Represents significant difference between Y-SFF and O-SFF at the selected mean; •*p* ≤ 0.05. ^#^Represents significant difference as compared to the LPS group at ^#^*p* ≤ 0.05

A robust and significant (over 10 folds) increase in intracellular ROS production was observed in macrophages treated with LPS suggesting a strong impact of LPS on cells (Fig. 6A). Conversely, a dose-dependent decrease in ROS levels was observed on account of SFF treatment in all cells (Fig. 6A). In particular, O-SFF treated cells appeared to be significantly more potent than Y-SFF treated cells in alleviating the LPS-induced increase in free radical formation such that no statistical difference in ROS production could be observed between control cells and O-SFF treated cells at 1% (v/v) concentration (Fig. 6A).

#### NO production

Similar to ROS production, NO levels recorded a significant and robust increase in LPS-treated cells as compared to the control (Fig. 6B). An age-dependent significant decrease in NO levels was also evident in both Y-SFF and O-SFF treated cells. Further, O-SFF treatment caused stronger inhibition of NO production in the wake of LPS stimulation than Y-SFF treatment (Fig. 6B).

#### Interleukins profile

**Fig. 7:**
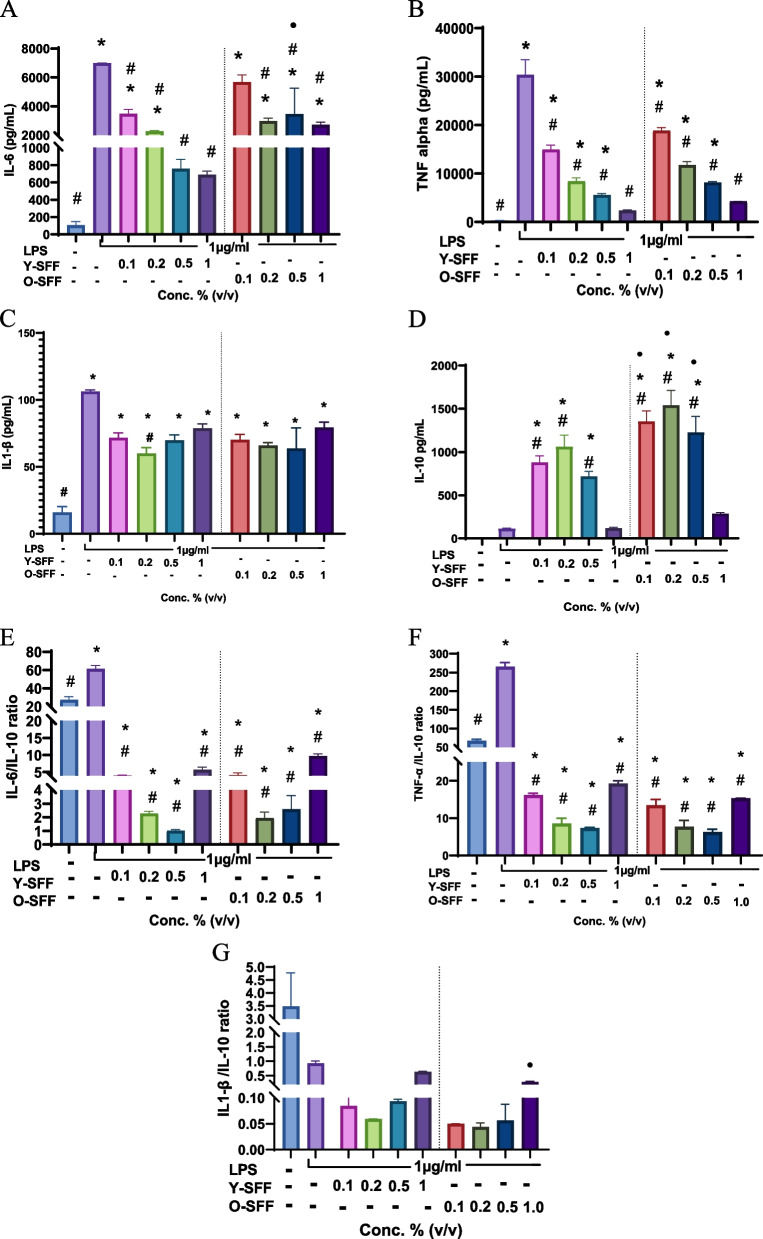
Immunomodulatory effects of Y-SFF and O-SFF treatment in attenuating LPS-induced inflammatory stress in macrophages (**A**) IL-6 (**B**) TNF-α (**C**) IL-1β (**D**) IL-10 (**E**) IL-6/IL-10 ratio (**F**) TNF-α /IL-10 ratio (**G**) IL-1β/IL-10 ratio production in macrophages at different concentrations. Values are mean ± S.D (*n* = 3). *Represents significant difference as compared to the control group; *p ≤ 0.05, **p ≤ 0.01, ***p ≤ 0.001, ****p ≤ 0.0001 •Represents significant difference between Y-SFF and O-SFF at the selected mean; •*p* ≤ 0.05, ••*p* ≤ 0.01, •••*p* ≤ 0.001, ••••*p* ≤ 0.0001. ^#^Represents significant difference as compared to the LPS group at.^#^*p* ≤ 0.05

As shown in Fig. 7, LPS treatment induced a massive inflammatory response in cells as evidenced by a significant increase in the levels of IL-6, TNF-α, and IL-1β. On the contrary, a mild and non-significant increase in anti-inflammatory cytokine IL-10 was observed in LPS-treated macrophages indicating a prevalent pro-inflammatory environment in LPS-treated macrophages. This was further confirmed by the ratio of pro-inflammatory cytokines to IL-10 which showed strong upregulation in response to LPS treatment (Fig. 7E-G). On the other hand, SFF treatment starkly reversed this scenario wherein a strong and significant inhibition of IL-6 and TNF-α was observed while a non-significant decrease in IL-1β was also apparent ultimately resulting in a significantly suppressed pro-inflammatory to anti-inflammatory interleukins ratio as compared to LPS treatment (Fig. 7). O-SFF treatment induced a significantly higher IL-10 response as compared to Y-SFF treatment which also resulted in a more balanced interleukins ratio.

#### Relative gene expression analyses

**Fig. 8:**
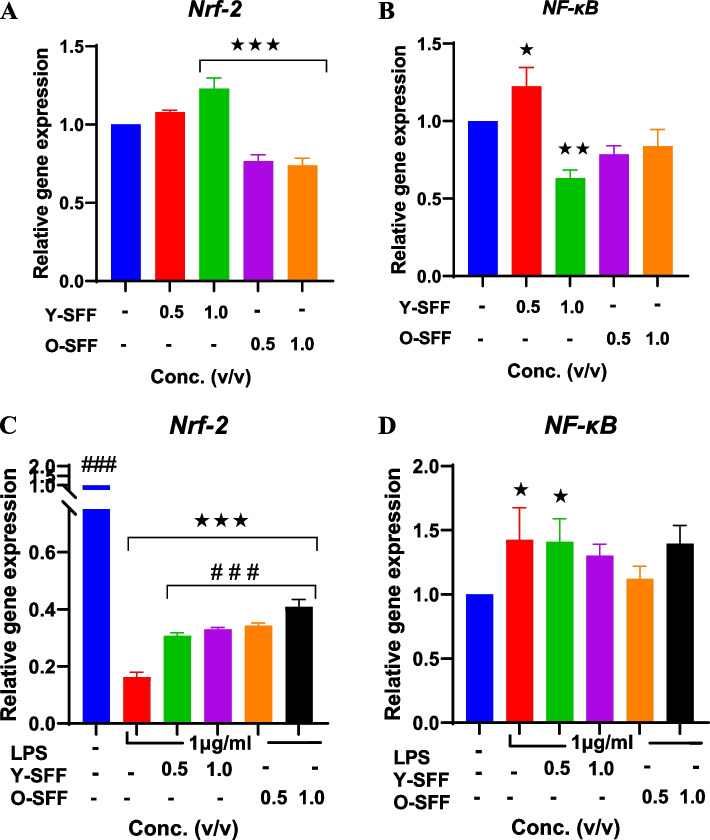
Influence of SFF on relative gene expression of *Nrf-2* and *NF-κB* during (**A**, **B**) immunostimulation and (**C**, **D**) during immunomodulation in response to LPS treatment. Values are mean ± S.D (*n* = 3). *Represents significant difference as compared to the control group; *p ≤ 0.05, **p ≤ 0.01, ***p ≤ 0.001, ****p ≤ 0.0001. ^###^Represents significant difference as compared to the LPS group at.^#^*p* ≤ 0.001

Relative gene expression of transcription factors *Nrf-2* and *NF-κB* were assessed in SFF samples at higher concentrations. In general, a suppressive effect of SFF treatment alone on both *Nrf-2* and *NF-κB* gene expression in macrophages was evident which was more robust in O-SFF treated cells (Fig. 8A, B). However, LPS treatment resulted in a massive and significant 6 folds decrease in *Nrf-2* expression while a significant increase was also noticeable in *NF-κB* (Fig. 8C, D). On the other hand, both Y-SFF and O-SFF significantly and strongly countered the LPS-induced decrease in *Nrf-2* expression of treated macrophages (up to 2.46 folds increase) while a non-significant decrease in *NF-κB* expression was also evident (Fig. 8C, D).

#### Antioxidant activity in SFF samples

**Fig. 9:**
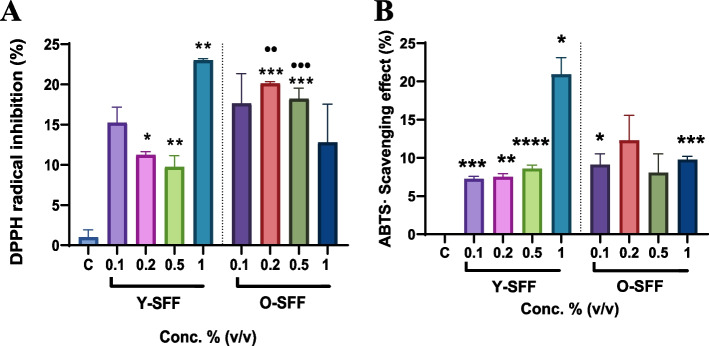
Estimation of inherent antioxidant capacity of Y-SFF and O-SFF (**A**) DPPH radical scavenging assay (**B**) ABTS radical scavenging assay. Values are mean ± S.D (*n* = 3). *Represents significant difference as compared to the control group, *p ≤ 0.05 **p ≤ 0.01, ***p ≤ 0.001, ****p ≤ 0.001 •Represents significant difference between Y-SFF and O-SFF at the selected mean; •*p* ≤ 0.05, ••*p* ≤ 0.01, •••*p* ≤ 0.001

Samples of Y-SFF and O-SFF were dissolved in PBS at concentrations already tested during cell culture and were further analyzed for their inherent antioxidant potency. Results showed significant DPPH radical inhibition by both Y-SFF and O-SFF as compared to the control, however, O-SFF appeared to be more potent than Y-SFF at all tested concentrations except at 1% (v/v) (Fig. 9A). Similarly, ABTS free radical scavenging activity of SFF appeared to be invariably higher than the control although no significant differences among Y-SFF and O-SFF could be observed (Fig. 9B).

#### GC/MS analysis of SFF

A total of 29 different volatile organic compounds (VOCs) were identified in Y-SFF while 26 different compounds were apparent in O-SFF through GC/MS analyses. These identified chemical constituents of Y-SFF and O-SFF with their retention time (RT), molecular weight (MW), peak area (%), and molecular formula are presented in Tables 1 and 2 respectively. Y-SFF documented the presence of Octamethyltrisiloxane (1), 2-Dimethylsilyloxypentane (2), Pyridinium, 1-(2-hydrazino-2-oxoethyl)-, chloride (3), Bicyclo[3.3.1]non-6-en-3-ol (4), 1,2-Dimethoxy-4-(1,3-dimethoxy-1-propenyl)benzene (5), Deoxyspergualin (6), 2,2-Diethylacetamide (7), Methyltris(trimethylsiloxy)silane (8), 3,7-Diacetamido-7H-s-triazolo[5,1-c]-s-triazole (9), 7,7,9,9,11,11-Hexamethyl-3,6,8,10,12,15-hexaoxa-7,9,11-trisilaheptadecane (10), Hexamethylcyclotrisiloxane (11), Ethyl(dimethyl)benzyloxysilane (12), Trisiloxane,1,1,1,5,5,5-hexamethyl-3,3-bis[(trimethylsilyl)oxy]- (13), Glycerol, 3TMS derivative (14), DL-Leucine, N-glycyl- (15), 11-(1-ethylpropyl) heneicosane (16), Tetradecane, 2,6,10-trimethyl- (17), 2,4,6-Tri-t-butylbenzenethiol (18), 1-Amino-2-[(2-bis-ethoxycarbonyl vinyl)amino]-4-chlorobenzene (19), Tris(tert-butyldimethylsilyloxy) arsane (20), Pyrazole[4,5-b]imidazole, 1-formyl-3-ethyl-6-á-d-ribofuranosyl (21), 1-Methyl-8-propyl-3,6-diazahomoadamantan-9-ol (22), 2-Myristynoyl pantetheine (23), 1-Hexadecanol, 2-methyl- (24), 7,9-Di-tert-butyl-1-oxaspiro(4,5)de ca-6,9-diene-2,8-dione (25), Panaxydol, TMS (26), Paromomycin (27), 2-Myristynoyl pantetheine (28), and Tetrasiloxane, 1,1,3,3,5,5,7,7-octamethyl (29).

**Table 1:** Chemical constituents identified in Y-SFF

**S. No**	**Retention time (RT, minutes)**	**Compound name**	**Area (%)**	**Mol weight (MW)**	**Molecular formula**
1	7.53	Trisiloxane, octamethyl	23.49	236	C8H24O2Si3
2	7.85	2-Dimethylsilyloxypentane	1.84	146	C7H18OSi
3	8.19	Pyridinium, 1-(2-hydrazino-2-oxoethyl)-, chloride	0.82	187	C7H10ClN3O
4	8.26	Bicyclo[3.3.1]non-6-en-3-ol	0.93	138	C9H14O
5	9.28	1,2-Dimethoxy-4-(1,3-dimethoxy-1-propenyl)benzene	2.94	238	C13H18O4
6	10.41	Deoxyspergualin	3.61	387	C17H37N7O3
7	10.72	2,2-Diethylacetamide	5.02	115	C6H13NO
8	11.25	Methyltris(trimethylsiloxy)silane	6.13	310	C10H30O3Si4
9	11.48	3,7-Diacetamido-7H-s-triazolo[5,1-c]-s-triazole	0.84	223	C7H9N7O2
10	12.25	7,7,9,9,11,11-Hexamethyl-3,6,8,10,12,15-hexaoxa-7,9,11-trisilaheptadecane	2.46	384	C14H36O6Si3
11	12.35	Hexamethyl cyclo-trisiloxane	3.99	222	C6H18O3Si3
12	13.26	Ethyl(dimethyl)benzyloxysilane	0.69	194	C11H18OSi
13	13.67	Trisiloxane,1,1,1,5,5,5-hexamethyl-3,3-bis[(trimethylsilyl)oxy]-	2.00	384	C12H36O4Si5
14	15.20	Glycerol, 3TMS derivative	4.02	308	C12H32O3Si3
15	15.88	DL-Leucine, N-glycyl-	0.60	188	C8H16N2O3
16	18.16	11-(1-ethylpropyl)heneicosane	1.32	366	C26H54
17	18.72	Tetradecane, 2,6,10-trimethyl-	0.69	240	C17H36
18	18.84	2,4,6-Tri-t-butylbenzenethiol	2.54	278	C18H30S
19	19.43	1-Amino-2-[(2-bis-ethoxycarbonylvinyl)amino]-4-chlorobenzene	0.74	312	C14H17ClN2O4
20	19.86	Tris(tert-butyldimethylsilyloxy) arsane	9.66	468	C18H45AsO3Si3
21	20.43	Pyrazole[4,5-b]imidazole,1-formyl-3-ethyl-6-á-d-ribofuranosyl	0.63	296	C12H16N4O5
22	20.58	1-Methyl-8-propyl-3,6-diazahomoadamantan-9-ol	0.72	224	C13H24N2O
23	20.69	2-Myristynoyl pantetheine	1.30	484	C25H44N2O5S
24	21.33	1-Hexadecanol, 2-methyl-	1.18	256	C17H36O
25	23.02	7,9-Di-tert-butyl-1-oxaspiro(4,5)deca-6,9-diene-2,8-dione	0.54	276	C17H24O3
26	23.30	Panaxydol, TMS	1.94	332	C20H32O2Si
27	25.50	Paromomycin	0.56	615	C23H45N5O14
28	25.79	2-Myristynoyl pantetheine	0.53	484	C25H44N2O5S
29	26.52	Tetrasiloxane,1,1,3,3,5,5,7,7-octamethyl-	1.19	282	C8H26O3Si4

**Table 2:** Chemical constituents identified in O-SFF

**S. No**	**Retention time (RT, minutes)**	**Compound name**	**Area (%)**	**Mol weight (MW)**	**Molecular formula**
1	7.54	Trisiloxane, octamethyl-	21.85	236	C8H24O2Si3
2	7.65	Acetamide, N-ethyl-	12.29	87	C4H9NO
3	7.85	2-Ethoxyethanol, TMS derivative	5.38	162	C7H18O2Si
4	8.20	Cystine	1.45	240	C6H12N2O4S2
5	8.26	3,7-Diacetamido-7H-s-triazolo[5,1-c]-s-triazole	1.04	223	C7H9N7O2
6	8.86	Pyridinium, dinitromethylide-	0.53	183	C6H5N3O4
7	8.97	Bicyclo[3.3.1]non-6-en-3-ol	1.47	138	C9H14O
8	9.28	t-Butyldiphenyl(prop-2-ynyloxy) silane	5.19	294	C19H22OSi
9	9.57	Pyridinium,1-(2-hydrazino-2-oxoethyl)-,chloride	0.88	187	C7H10ClN3O
10	10.42	Mannosamine	4.03	179	C6H13NO5
11	10.51	Cephaloridine	1.42	415	C19H17N3O4S2
12	10.72	2,2-Diethylacetamide	8.51	115	C6H13NO
13	11.25	Methyltris(trimethylsiloxy) silane	10.07	310	C10H30O3Si4
14	11.60	Silane, triethyl(2-phenylethoxy)-	1.72	236	C14H24OSi
15	12.24	7,7,9,9,11,11-Hexamethyl-3,6,8,10,12,15-hexaoxa-7,9,11-trisilaheptadecane	2.54	384	C14H36O6Si3
16	12.35	2,4,6-Cycloheptatrien-1-one,3,5-bis-trimethylsilyl-	4.64	250	C13H22OSi2
17	13.67	Trisiloxane,1,1,1,5,5,5-hexamethyl-3,3-bis[(trimethylsilyl)oxy]-	1.76	384	C12H36O4Si5
18	15.20	Glycerol, 3TMS derivative	2.02	308	C12H32O3Si3
19	17.85	Paromomycin	0.46	615	C23H45N5O14
20	18.80	Pterin-6-carboxylic acid	0.43	207	C7H5N5O3
21	19.86	Thieno[2,3-c]furan-3-carbonitrile, 2-amino-4,6-dihydro-4,4,6,6-tetramethyl	0.75	222	C11H14N2OS
22	22.96	Pyrazole[4,5-b]imidazole,1-formyl-3-ethyl-6-á-d-ribofuranosyl	0.39	296	C12H16N4O5
23	24.18	Androstane-11,17-dione,3-[(trimethylsilyl)oxy]-,17-[O-phenylmethyl)oxime],	0.52	481	C29H43NO3Si
24	30.52	4-Dehydroxy-N-(4,5-methylenedioxy-2-nitrobenzylidene)tyramine	0.42	298	C16H14N2O4
25	32.74	Thieno[2,3-c]furan-3-carbonitrile,2-amino-4,6-dihydro-4,4,6,6-tetramethyl	0.47	222	C11H14N2OS
26	34.62	Carbamic acid	0.39	353	C20H23N3O3

On the other hand, O-SFF was characterized by the presence of Trisiloxane, octamethyl- (1), Acetamide, N-ethyl- (2), 2-Ethoxyethanol, TMS derivative (3), Cystine (4), 3,7-Diacetamido-7H-s-triazolo[5,1-c]-s-triazole (5), Pyridinium, dinitromethylide (6), Bicyclo[3.3.1]non-6-en-3-ol (7), t-Butyldiphenyl(prop-2-ynyloxy) silane (8), Pyridinium, 1-(2-hydrazino-2-oxoethyl) chloride (9), Mannosamine (10), Cephaloridine (11), 2,2-Diethylacetamide (12), Methyltris(trimethylsiloxy) silane (13), Silane, triethyl(2-phenylethoxy) (14), 7,7,9,9,11,11-Hexamethyl-3,6,8,10,12,15-hexaoxa-7,9,11-trisilaheptadecane (15), 2,4,6-Cycloheptatrien-1-one, 3,5-bis-trimethylsilyl (16), Trisiloxane, 1,1,1,5,5,5-hexamethyl-3,3-bis[(trimethylsilyl)oxy] (17), Glycerol, 3TMS derivative (18), Paromomycin (19), Pterin-6-carboxylic acid (20), Thieno[2,3-c]furan-3-carbonitrile, 2-amino-4,6-dihydro-4,4,6,6-tetramethyl (21), Pyrazole[4,5-b]imidazole, 1-formyl-3-ethyl-6-á-d-ribofuranosyl (22), Androstane-11,17-dione, 3-[(trimethylsilyl)oxy]-17-[O-phenylmethyl)oxime] (23), 4-Dehydroxy-N-(4,5-methylenedioxy-2-nitrobenzylidene)tyramine (24), Thieno[2,3-c]furan-3-carbonitrile, 2-amino-4,6-dihydro-4,4,6,6-tetramethyl (25), and Carbamic acid (26).

## Discussion

Disrupted crosstalk between the gut microbiome and immune cells, including macrophages, is a key mediator of several inflammatory disorders of the gut [23–25]. Macrophages, in particular, are emerging as potential therapeutic targets for the management of inflammation and gut injury [26]. In addition, the beneficial effects of FMT therapy have been related to the modulation of macrophage effector functions [27, 28]. However, considering the limitations and challenges associated with FMT, the use of SFF is recently being recognized as an alternate approach for managing gut inflammatory disorders [14, 29]. The present study thus evaluated whether SFF has the potency to stimulate as well as modulate macrophage functions that may be useful for maintaining cellular immune homeostasis and resisting inflammatory disorders respectively. Our results showed that regardless of the donor mice age, SFF treatment strongly stimulated macrophage activity as determined by cellular morphological changes, NO production, and respiratory burst capacity. When stimulated, macrophages display characteristic polygonal and dendritic-like morphology and considerably increased production of cellular ROS along with NO that primes the macrophages for impending bactericidal activities [22, 30, 31]. SFF treatment in the present study also induced macrophage pro-inflammatory response particularly characterized by increased levels of IL-6 and TNF-α while a slightly improved inflammatory homeostasis was evident in Y-SFF treated cells as compared to O-SFF. Previous studies have reported that gut probiotic bacteria metabolites can stimulate macrophage functions characterized by increased respiratory burst, phagocytic activity, ROS production, and increased pro-inflammatory cytokines [32, 33]. However, studies directly exploring the role of FMT or SFF on macrophage stimulation in the absence of any external inflammatory threat are extremely rare. In this regard, similar to our observations, a recent report indicated that SFF of healthy donors can induce a slight increase in pro-inflammatory IL-1β expression in M1 macrophages differentiated from CD14 + monocytes, although no effect on TNF-α levels could be observed [34]. However, to the best of our knowledge, the present study is the first systematic report examining the immunostimulatory effects of SFF on mature resting macrophages. Our results also demonstrated near similar immunostimulatory attributes of both Y-SFF and O-SFF on macrophages. It is pertinent to note here that the impact of age on gut microbial composition and diversity is not direct or linear per se but is subject to diet, genetics, sex, and environmental regulation. Several studies have reported that gut microbiota composition and diversity remains diverse and balanced in healthy elderly with little impact of age while age-dependent loss of gut microbial diversity is fairly common in elderly suffering from co-morbidities [35–38]. Regardless, the functional implications of any age-dependent gut microbiota changes (and metabolome thereof) are yet to be completely understood, and in the wake of our results, it appears that the overall fecal metabolome functional diversity may not necessarily be modulated with age at least in healthy elderly.

The gut represents a large reservoir of gram-negative bacteria that constantly shed their LPS into the colonic lumen which is traditionally considered a source of pro-inflammatory stimuli [39]. In our study, we observed characteristically strong upregulation of all inflammatory and oxidative stress markers in macrophages on account of LPS stimulation. Further, a very strong suppression in *Nrf-2* gene expression was also evident in LPS-treated cells which can result in aggravation of pro-inflammatory cytokines production by activating *NF-kB* [40–42]. Interestingly, on comparison of LPS treatment with immunostimulatory effects of both Y-SFF and O-SFF in resting macrophages; it was evident that SFF application did not adversely augment macrophage functions. This is because a multifold increase in various tested oxidative and inflammatory stress parameters in LPS-treated cells was observed as compared to SFF treatment alone thereby suggesting that SFF exposure may not have caused unwarranted exacerbation in the inflammatory phenotype, but indeed primed the macrophages for robust effector functions. The apparent lack of activation of either *NF-κB* or *Nrf-2* expression in resting macrophages also supports these observations. In addition, previous studies have shown that natural agents, including probiotic bacteria, can induce macrophage stimulation such that it results in improved effector functions in the wake of external inflammatory threats [31, 43]. In the present study, pre-treatment with SFF demonstrated very strong dose-dependent anti-inflammatory attributes in mitigating the effects of LPS characterized by robust upregulation of IL-10 expression and concomitant suppression of TNF-α, IL-6, and IL-1β levels. Further hyporesponsiveness to LPS was evidenced by decreased NO and ROS production in SFF-treated macrophages. Although FMT therapy can protect against inflammatory bowel diseases by suppressing immune cell activation, however, studies directly assessing SFF for their anti-inflammatory efficacy relating to macrophages and/or other immune cells are extremely rare [27, 28, 44–46]. In a study based on gut inflammation, it was observed that fecal-derived luminal factors from patients of ulcerative colitis were less potent in alleviating LPS-induced inflammation as compared to fecal luminal factors derived from the healthy control group in monocyte-derived macrophages in vitro [34]. Our study also indicated that similar to immunostimulatory analyses, the age of the donor mice had little effect on SFF-mediated immunomodulatory activity against LPS in macrophages. If at all, slightly improved effects of O-SFF application in attenuating LPS-induced oxidative stress were evident which further suggests little age-dependent correlation in the efficacy of fecal metabolome.

The apparent pharmacological effects of SFF could be attributed to the presence of a diverse mixture of alkanes, organosulfur compounds, amides, alkaloids, antimicrobial compounds, lipids, and amino acids especially since a modest inherent antioxidant activity was recorded in both Y-SFF and O-SFF which correlated with improved cellular *Nrf-2* gene expression and redox stress in LPS treated cells. We observed several unique compounds in Y-SFF and O-SFF along with a few similar constituents although the overall profile of classes of chemical constituents did not appear to change drastically. This lack of strong characteristic changes in the chemical profile of Y-SFF and O-SFF is further suggestive of observed limited differences in their cellular modulatory functions in the present study. These observations are also in agreement with previous studies that characterized volatile organic compounds in the feces of both animals and humans [47, 48]. In addition to metabolites, the fecal filtrate is also enriched in various species of viruses including bacteriophages, and the efficacy of the fecal virome in directly suppressing gut inflammatory disorders and immunoregulation is rapidly emerging which may also have contributed to the apparent immunomodulatory effects of SFF observed in this study [49–51]. Concerning LPS stimulation work in the present study, it is pertinent to note that small levels of gut-derived LPS may also be present in the tested SFF samples as recorded previously [52]. However, although gut-derived LPS has traditionally been viewed as a pro-inflammatory agent, emerging research is challenging this viewpoint. It has been demonstrated that total freely circulating LPS originating from a healthy gut is anti-inflammatory and immuno-inhibitory due to its structural differences as compared to *E. coli*-based LPS which is often used in in vitro stimulation studies [53]. In particular, members of the *Bacteroides* genus of the gut microbiome have structurally and functionally distinct variants of LPS that have been shown to exert immune-suppressive effects as compared to *E. coli*-based LPS [54–56]. Therefore, it is plausible that the known anti-inflammatory attributes of FMT therapy or fecal-derived luminal factors could be positively associated with the type and levels of gut-derived LPS present. Our results also support this notion and suggest that any LPS present in the SFF may have directly contributed to its apparent robust anti-inflammatory response although further specific studies in this regard are recommended for a causal understanding.

## Conclusions and outlook

The association between gut microorganisms and human physiology is deep and intricate. The gut could be considered a large bioreactor wherein complex dietary metabolites are broken down by the gut microbiota, and novel metabolites are produced that can significantly affect human physiology both in health and disease [57, 58]. Feces are essentially representative of the fermented metabolome of the organism and thus have been considered useful non-invasive markers of gut health and disease [59, 60]. Fermentation by the unique microorganisms in the gut can significantly alter the metabolic characteristic of ingested dietary factors and confer superior beneficial effects as also observed in the case of *Kopi luwak* coffee beans [61, 62]. Further, the applicability of feces as sources of therapeutic metabolites can be justified when used in the SFF form that negates any harmful microorganisms except viruses. Due to the growing concern over the pertinence and safety of FMT, a considerable interest in developing an alternate SFF-based approach is rationalized. In this regard, the present work substantially enhances our therapeutic understanding of SFF. Our study suggests that bacterial metabolites or their components as well as bacteriophages/viruses present in the SFF have strong pharmacological effects that could mediate the classical health benefits of FMT transfer. Thus, SFF could be used as an alternate therapeutic system especially against inflammatory disorders. Further, since components of SFF are more likely to come in contact with cells of the gut mucosa and even enter systemic circulation [63]; they may present promising therapeutic agents against chronic inflammatory disorders even distal to the gut. Therefore, our study recommends the exploration of the use of SFF not only in mediating the effects of FMT but also as a novel therapeutic agent against chronic inflammatory conditions. In addition, it would be interesting to see how the modulation of the gut microbiome by nutritional factors, antibiotics, or in disease condition impacts the efficacy of SFF [64]. As such, further studies based on in vivo models are recommended for a deeper understanding of the potential therapeutic benefits of SFF.

## Data Availability

All data generated or analyzed during this study are included in this article.
